# Advantages of the Use of Glycerine in Surgery

**Published:** 1860-05

**Authors:** 


*Advantages of the Use of Glycerine in Surgery.*—M. Demarquay, a distinguished hospital surgeon of Paris, has used, and recommends, glycerine in ulcers and fistulous tracts, along which latter it should be injected to fulfil the following indications—viz., to diminish excessive suppuration, cleanse the secreting surfaces, modify the noxious properties of the pus, prevent the stagnation of fluids, or simply to excite the pyogenic membrane, and bring about cicatrization.

Glycerine may be advantageously used in deep abscesses connected with diseased bone, and in such cases the author combines glycerine with iodine, because the former is, alcohol excepted, the best solvent of the latter, and penetrates very powerfully, reaching to a great depth. Glycerine may also be employed in the dressing of scorbutic, scrofulous, syphilitic, and atonic ulcers, either alone or as preparatory to another kind of treatment—viz: compression with straps of adhesive plaster. When used for ulcerated chilblains, glycerine should be extremely pure, because it is apt, when not quite free from foreign substances, to excite very painful inflammation.—*Drug Cir.*

---

There exists a substance possessed of powerful and definite properties, and having the remarkable property of restoring to health, or, at all events, of greatly relieving the disordered nervous system of persons suffering from chronic alcoholism: the medicinal agent in question acting efficaciously in cases where the principal symptom may be either sleeplessness or hallucinations or trembling, or any other, and this substance is *oxide of zinc.—Ibid.*

